# Developing a full-scale shaking codend to reduce the capture of small fish

**DOI:** 10.1371/journal.pone.0280751

**Published:** 2023-01-23

**Authors:** Vang Y. Nguyen, Shannon M. Bayse, Zhaohai Cheng, Paul D. Winger, Harold DeLouche, Gebremeskel Eshetu Kebede, George Legge

**Affiliations:** Fisheries and Marine Institute, Memorial University of Newfoundland, St. John’s, NL, Canada; COISPA Tecnologia & Ricerca - Stazione Sperimentale per lo Studio delle Risorse del Mare, ITALY

## Abstract

To reduce the retention of undersized fish in the redfish (*Sebastes* spp.) trawl fishery in the Gulf of St. Lawrence, Canada, we developed a full-scale shaking codend. The shaking codend uses a mechanical stimulating device, an elliptical-shaped piece of polyvinyl chloride canvas, attached to the posterior of a T90 codend that generates a lifting force with respect to drag, causing a ‘shaking motion’. A shaking codend could stimulate fish movement and increase contact probability, both of which could increase the escape of small redfish out of a codend, especially when combined with a codend that maintains mesh openings. The movement and fishing characteristics of a shaking codend (T90 codend with canvas) relative to a T90 codend (without canvas) were tested in a flume tank and field experiment. In the flume tank test, the shaking codend had a peak-to-peak amplitude (i.e. the distance the codend moves from the lowest to highest depth) > 24 cm higher than the T90 codend for each velocity tested (1.0–1.8 kt), higher amplitude ratio, and a higher period (1 revolution) that gradually decreased with increasing velocity. The total acceleration (m s^-2^) and drag forces (kgf) estimated for the shaking codend were significantly higher than the T90 codend across all flow velocities. The results from the field experiment, considered preliminary due to a small sample size, showed that the shaking codend significantly reduced the capture of small redfish (< 21 cm) and the best fit model did not need to consider contact probability which was necessary for the non-shaking T90 codend. Overall, the dynamics of the movement of the codend was described and could be potentially used as an effective technique to reduce the catch of small redfish, and perhaps in other trawl fisheries to reduce the catch of small fish.

## Introduction

Discards are the portion of the catch consisting of unwanted species that are either too small or have no market value and are thrown overboard at sea after capture [1, 2]. Estimating and reducing discards in the catch of marine fisheries, particularly in commercial fishing, has been the subject of much concern by fisheries management, fishers, and researchers in recent decades. Estimated annual discards in global marine fisheries during 2010–2014 were around 9.1 million t, occupying approximately 10.8% of the total annual catch [3]. The amount of discard varies according to region and gear type. For example, in the Northeast Atlantic and Northwest Pacific, the annual discards occupied 39% of annual totals, with 33% contributed by bottom trawl fisheries [3].

Discarding from commercial fisheries is expected to impact marine ecosystems and stock management globally. High levels of discards in marine fisheries threatens sustainable fisheries by inducing unnecessary fishing mortality, which is considered a waste of natural resources [1, 4]. Additionally, discard problems make fisheries management designs and execution difficult [5]. Thus, in recent years, fisheries have been managed with discard quotas, effort regulation, no-discard regimes, and selective fishing to reduce discards [1, 5].

In the Gulf of St. Lawrence, CA, two redfish species are commercially harvested, deepwater redfish (*Sebastes mentella*) and Acadian redfish (*S*. *fasciatus*), and are typically considered together as redfish (*Sebastes* spp.) [6, 7]. These species are slow growing, late maturing, and long-lived and thus are susceptible to overfishing [8]. In 1995, the Gulf of St. Lawrence (Unit 1) redfish fishery was placed into moratorium, and only a small (2,000 t year^-1^) index fishery has taken place since 1999 [7]. However, due to recent strong recruitment events, there is a large redfish biomass now found in the Gulf of St. Lawrence, which will lead to a reopening of the commercial fishery [7].

Current conservation measures for the redfish fishery include a small fish protocol, bycatch protocol, minimum landing size of 22 cm, and the use of a mesh opening of 90 mm [7, 9]. Recent studies have attempted to develop trawls to further reduce the catch of small redfish [10, 11] and a T90 codend was shown to effectively reduce the capture of undersized redfish [11]. Considering that the high proportion of undersized redfish captured led to the redfish fishery moratorium in the 1990s [12], continued research should be applied to further reduce the capture of small redfish in the commercial fishery.

Discards can be reduced by the addition of a so called bycatch reduction device (BRD), where bycatch is the retention or discarding of a non-target species or specific sizes of target species [13]. A BRD is the addition of a device to a fishing gear (e.g. grid, large-mesh panel, etc.) that reduces the capture of unwanted animals or animal sizes [14]. Recently, several studies have investigated the use of active stimulating devices (ASDs) as, or in conjunction with, a BRD to increase the escape of undersized fish in trawls. ASDs have been developed to encourage fish to approach netting, BRDs (e.g. square mesh panel), or an area of a fishing gear (typically trawl) to increase the likelihood of escape. Thus, an ASD stimulates fish to react to a moving object (e.g. rope, trawl panel) increasing the likelihood of contact and escape through a BRD, mesh, or other opening [15–17].

Past ASD experiments include a tank experiment by Kim and Whang [16] that found that the retention of juvenile red seabream was reduced below 20% when an array-rope stimulation was introduced in the codend. This type of ASD changed how fish reacted in the codend by increasing an erratic response, which encouraged individuals to approach the netting and escape from the codend. Herrmann et al. [16] and Cuende et al. [18] have investigated the increase in escape of cod through a square-mesh panel using fluttering ropes with floats mounted to the bottom panel of the codend in the Baltic Sea. Additionally, Grimaldo et al. [15] showed that mechanical stimulation can trigger escape behaviors for haddock in the Barents Sea demersal trawl fishery. Kim [19, 20] used a circular piece of canvas at the posterior of the codend to form a cap which generates lifting force with respect to drag, inducing a “shaking” movement of the codend. This movement was shown to lead to an increased escape rate for small fish through the codend meshes. Escape rates of juvenile fish were observed to increase by 22–30% when compared with a codend without the canvas [19, 20].

Alternative codend size-selectivity research into increasing mesh size, changing mesh shape, and net construction has been effective, in certain cases, at reducing the capture of small fish in trawls [21–23]. Several studies have modified diamond mesh codends to improve the size selectivity for groundfish [24], which includes redfish fisheries [10, 25]. A diamond mesh codend rotated 90° in the transversal direction, called a T90 codend, has been shown to significantly reduce the capture of small roundfish [24, 26, 27]. Cheng et al. [11] applied three T90 codends in the Canadian redfish fishery and reduced the capture of small fish.

The objective of this study was to develop a shaking codend to reduce the capture of small redfish. For the first time, a T90 codend was used in conjunction with a shaking codend to potentially aid the escape of small redfish. Since T90 meshes remain open during hauling, as opposed to diamond meshes that close under tension of the catch, they can lead to a reduction in capture of small round fish [24, 26] and combining bycatch reduction technology has been shown to even further reduce bycatch [28]. The moving dynamics of adding a canvas to the posterior of a commercial codend was described and compared to a codend without a canvas in a flume tank test. Additionally, a full-scale sea trial was attempted for the first time using a shaking codend using commercial gear in a commercial fishing scenario, comparing the catches with and without the canvas using a covered codend technique.

## Materials and methods

### Flume tank experiment

A four-panel codend constructed of double-braided polyethylene netting (nominal 4.6 mm ∅) with meshes configured as T90 (nominal stretched inside mesh opening of 90 mm) was used for flume tank tests. The codend was attached to an extension made of the same netting, 3 meshes made up the selvedges, and the riblines were Quicklines (DynIce DuxTM, Dyneema, 18/22 mm ∅) 5% shorter than the selvedges.

**Fig 1:**
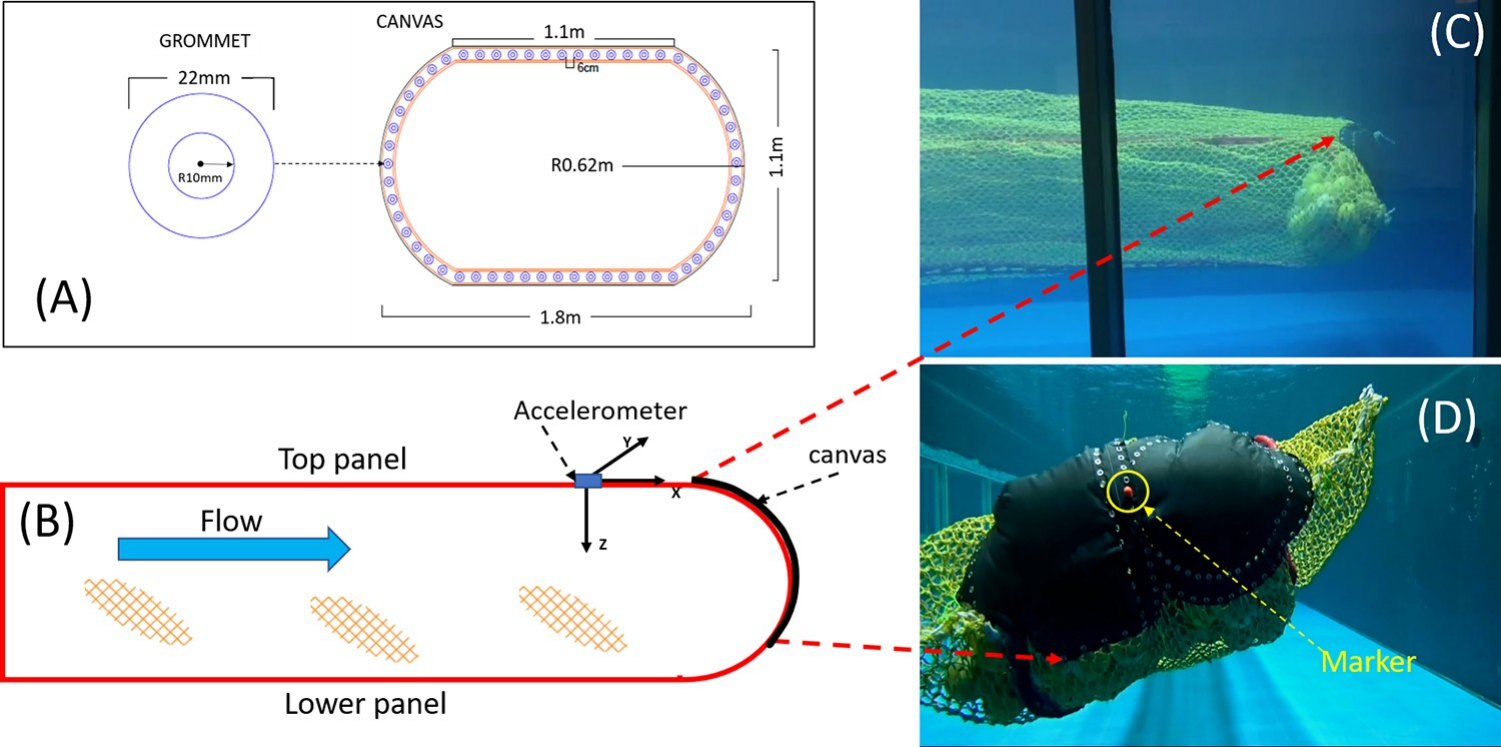
Canvas specification and location on codend. (A) detailed plan of canvas; (B) side-profile schematic illustration of the anterior section of the codend; (C) side-profile photo of the anterior section of the codend during flume tank tests; and (D) photo of the posterior of the codend during flume tank tests.

For experimental treatments, a black, elliptical-shaped canvas (Polyvinyl chloride, R 62 cm) was attached to the posterior of the codend, positioned slightly over the top of the codend similar to Kim [19] (Fig 1). The canvas was 1.8 m in length, 1.1 m wide, and had a radius of 0.62 m. For tank tests, two canvases were attached and overlapped to make up these dimensions, for sea trials, one canvas was used; for both cases, the perimeter of the canvas was the same. The final version had a total of 58 grommets of 22 mm dimensions included around the canvas’s edge for connecting to the end of the codend, and the distance between consecutive grommets was 6 cm (Fig 1A). The canvas was attached to the codend via plastic ties through the grommets on previously marked meshes for consistency.

Codend movement was quantified in a flume tank located at the Centre for Sustainable Aquatic Resources, Fisheries and Marine Institute of Memorial University of Newfoundland, Canada, between 02 and 27 April 2019. The flume tank test area is 22.3 m long, 8.0 m wide, and 4.0 m deep, and can maintain water velocities up to 1.8 kt, and the side observation window is 20 m long x 3 m in height [29].

For testing, the codend was attached to a towing mast by four steel bridles (2 m) and a steel ring (140 cm ∅) at the anterior of the codend extension. Trawl floats (*n* = 80; 20 cm ∅; Pescaflot N-90, Castro, Donostia, Gipuzkoa, Spain) were added to the codend to simulate accumulated catch that weighed approximately 350 kg (estimated by volume) following Cheng et al. [30]. Each float had six holes drilled (2.6 cm ∅) through it to balance its weight and buoyancy. The total weight of floats in water was 0.0 kg. Shaking and T90 codend experiments were tested separately with five flow velocities, from 1.0 to 1.8 kt with a 0.2 kt increment. The duration of each flow velocity tested was performed for 30 min, except for one iteration of the T90 codend, which was tested for 14 min and 45 sec at the flow velocity of 1.0 kt.

The moving angle (°) of the shaking codend for each flow velocity was measured. The moving angle is defined by the angle formed between the vertical axis and the direction that the shaking codend moved in the vertical plane which was perpendicular to the flow direction in the flume tank. The moving direction was determined by tracking the center of the canvas. Thus, a marker (small red ball) was attached to the center of the canvas (Fig 1D), and its movement was recorded using the camera positioned at the end of the flume tank looking toward the posterior of the codend. For the moving angle analysis, a 10 min and 30 sec video recording was made and subsampled at each flow velocity. HITFILM 4 EXPRESS software (FXhome, Norwich, England) was used to track the movement of the red marker. The tracking was performed frame-by-frame until the end of each subsample. The moving angle was measured for every 1 min of tracking using ImageJ software (http://rsb.info.nih.gov/ij).

Codend acceleration (m s^-2^), the rate of change in the velocity of the codend movement, was measured by an accelerometer (HOBO, UA-004-64 Pendant G Data logger, Bourne, MA, USA). Acceleration was recorded every second in three directions, X- (anterior to posterior), Y- (port to starboard) and Z-direction (top to bottom) (Fig 1B). The codend acceleration is represented by the total acceleration (TA (m s^-2^)) [31, 32], which is calculated by summing accelerations from three directions using the equation:

$$TA=\surd ({X)}^{2}+{\left(Y\right)}^{2}+{\left(Z\right)}^{2}$$ (1)

where X, Y, Z are acceleration values recorded in X, Y, Z-direction, respectively.

Drag (kgf) was measured using a 500 lb load cell (Model-No. 31, Honeywell, USA). The load cell was attached to a connection which consisted of four steel bridles with a towing mast for the purpose of measuring drag forces of each codend separately at flow velocities between 1.0 and 1.8 kt.

The distance (cm) that the codends moved vertically was described using a depth sensor (HOBO, U20L-02 Water Level Data Logger, Bourne, MA, USA). Depth was recorded every second. The logger was attached to the middle of the side panel, right before the front edge of the canvas. The change in depth allowed each codend to be characterized by amplitude ratio and period [19, 20]. The amplitude ratio was defined by the peak-to-peak amplitude divided by the length of the side panel. The peak-to-peak amplitude describes the distance the codend moves from the lowest to highest depth. The lowest and highest depth (peak) was determined using the find_peaks function in the ggpmisc package [33] of R Statistical Software [34]. The length of the side panel was measured from a side-view video using ImageJ software. A total of 20 frames were randomly chosen for the measurement, and the mean length of the side panel was 99.6 cm (Standard Error of the Mean (SEM) = 0.5 cm). The period was defined as the time of one oscillation, derived from the peak-to-peak analysis.

### Flume tank experiment analysis

A simple linear regression was used to determine the relationship between moving angle and flow velocity using the lm function in base R. Total acceleration, drag, and amplitude ratio were analyzed with a multiple regression using the lm function, where independent variables included codend, flow velocity, and their interaction term. The best model was selected based on the minimum Akaike information criterion value [35] with a correction for small sample sizes (AICc) and was calculated using the AICctab function in the bbmle package [36]. A model that had a ΔAICc < 2 was considered the best model. A post hoc test was performed with the TukeyHSD function to compare differences between codends and flow velocities following an analysis of variance (ANOVA; aov function).

Period data was collected every second, thus could be considered count-based (by individual second) and were analyzed initially with a Poisson distributed model using the glm function. The dependent variable was period, independent variables were codend and flow velocity, and their interaction term. Dispersion was estimated with the DHARMa package [37], which approximates dispersion with simulations. If the resultant model was equidispersed (dispersion ~ 1.0), then the analysis continued with the Poisson distributed model, if overdispersed (dispersion > 1.0) then the model would be fit with a negative binomial distribution, and if underdispersed (dispersion < 1.0) then the model would be fit with a quasi-Poisson model. Model selection followed the AICc methods described above, unless the model was underdispersed. In that case, QAIC was used for model selection following the methods described in Bolker [38]. A post hoc test, general linear hypothesis test–Tukey all-pair comparisons (glht function in the multcomp package; [39] was used to compare period between codends and flow velocity.

### Sea trials

This study did not involve any endangered or protected species. Experimental fishing was performed on a commercial fishing vessel *F/V Lisa M* (overall length 19.8 m; gross tonnage 122.5 t; engine power 700 horse power; 1 hp = 746 W) in accordance with the experimental fishing license granted by Fisheries and Oceans Canada (NL-5596-19). The license required that all redfish catches be landed.

**Fig 2:**
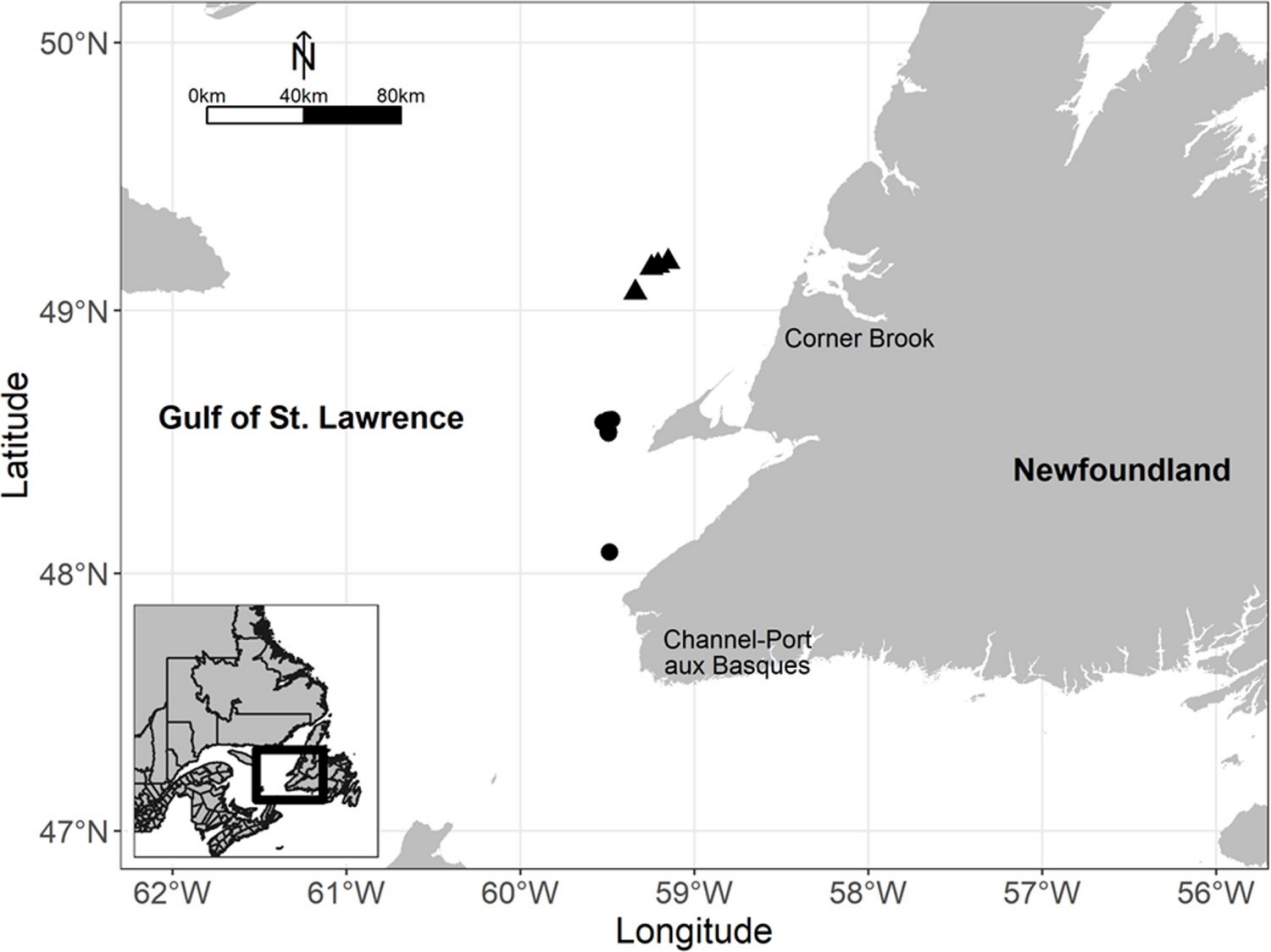
Map of fishing trials in the Gulf of St. Lawrence, Canada. Black points represent locations of shaking codend hauls, black triangles represent locations of T90 codend hauls. Reprinted from GADM under a CC BY license, with permission from [https://gadm.org], original copyright [2022].

Trials were conducted off the west coast off Newfoundland in the Gulf of St. Lawrence, Canada between 16 July and 02 August 2019 (Fig 2). Fishing locations were determined by the captain. Gear performance (towing velocity, duration, warp length, and door spread) was recorded for each haul. All hauls were fished during the day. Haul durations for the experiment were shorter than typical for the fishery. Generally, at high catch rates a tow of 1–2 hr is typical and longer with lower catch rates (personal observation). Short tow durations can be successful however since redfish can be highly congregated. Our tows were short for a few reasons, high catch volumes in both a codend and cover were difficult to handle at the same time and we had to use caution while fishing on limited quotas. Ultimately, our tow times were based on the goal of having similar catch volumes per tow, which can change throughout the day with redfish dial migration.

The codend described in the tank experiment was attached to a commercial groundfish bottom trawl for sea trials and mesh sizes were measured using an ICES OMEGA mesh gauge; 40 meshes measured per codend while wet [40]. The trawl was a high opening balloon trawl described in Cheng et al. [11]. The fishing line and headline of the trawl were 44.5 m and 40.2 m in length, respectively. The trawl belly sections were constructed with the same netting, 170 mm diamond PE twine with ∅ (diameter) from 3.5 to 4.0 mm. The headline consisted of 132 floats (20.3 cm ∅). The trawl was equipped with rockhopper footgear and the diameter of rubber discs ranged between 36 mm and 41 mm ∅. The trawl was rigged with a pair of low-aspect trawl doors, (Injector Door Limited, Søvik, Norway), which were 4 m^2^ in area. The door spread was recorded using acoustic sensors during trawling (Notus Electronics Ltd., St. John’s, Newfoundland and Labrador, Canada).

We applied the covered codend method to estimate the difference in size selectivity between shaking and T90 codend [41]. A 39.7 m long two-seam cover was used for both tested codends, constructed with single 2.5 mm PE twine panels and attached to the end of the extension. The nominal mesh size of the cover codend was 50 mm. To prevent the cover from masking the codend, flexible kites were used following Grimaldo et al. [42]. A total of 29 kites were attached to the cover net.

The codend movement at sea was recorded using a depth sensor capable of going to deep depths (Starmon TD, Star Oddi, Garðabær, Iceland). Depth data was only considered 1 min after the start of the haul and 1 min before haul back. The method applied to determine the amplitude ratio was similar to what was used in the flume tank test, side panel length used to determine the ratio was the same as what was used in the flume tank test. However, vessel depth recordings indicated that bottom depths were continually changing, thus, subsamples of recorded depths were taken to estimate amplitude ratio and period when depths did not change greater than 0.5 m over the considered time interval. In cases of multiple highest or lowest points (of the same value), the middle point between readings was chose to correspond to the average time recorded between these points. Cheng et al. [30] showed that the tested T90 codend only had a minimal change in water flow inside the codend with the addition of the covered codend (0.05–0.10 m s^-1^) during a flume tank test, thus the effect of the cover was considered minimal on codend movement.

Captured redfish from the codend and the cover were landed on the deck separately. A random subsample was taken for lengths measurements for both the codend and cover. All captured redfish were weighed. Redfish fork lengths measurements were made to the nearest centimeter, and species was determined by anal ray count [43] and reported as *S*. *mentella* in Cheng et al. [11]. Redfish catches were only considered here, bycatch was reported in Cheng et al. [11].

### Sea trials analysis

Size selectivity analysis was performed using SELNET [44] and followed techniques previously described in Bayse et al. [24], Cheng et al. [10], and Einarsson et al. [45]. Fish were assumed to enter the codend and either be retained in the codend or escape into the cover. This enables catch data to be considered as a binomial distribution. The function *rj(l)* was used to estimate the probability of a fish of length *l* in haul *j* being retained in the codend, and thus estimate the values of this function for all relevant redfish sizes. The retention probability estimation was carried out for all observed size classes (cm^-1^) and was expected to vary between hauls [46]. Thus, hauls were pooled between treatments to describe the length-dependent probability averaged over hauls, *rav(l)* [44]. Since more than one model was applied, *rav(l*,*v)* was used to describe the length-dependent probability retained in the main codend averaged over hauls, where *(v*) is the model parameters. Model parameters were estimated using maximum likelihood estimation. If the model can describe the data well, Eq (2) was used to maximize the likelihood of data describing the number of fish retained in the codend *(nR*_*jl*_*)* and cover *(nE*_*jl*_*)*.


$$-\sum_{j=1}^{m}\sum_{l}\left\{\frac{{nR}_{jl}}{{qR}_{j}}\times ln\left(r_{av}(l,\nu )\right)+\frac{{nE}_{jl}}{{qE}_{j}}\times ln\left(1.0-r_{av}(l,\nu )\right)\right\}$$ (2)

Subsample factors were *qR*_*j*_ and *qE*_*j*_ for the codend and the cover, respectfully.

A total of eight size selectivity models were tested to describe *r*_*av*_*(l*,*v)* for each codend (Eq 3). The first four models, Logit, Probit, Gompertz, and Richard, are classical size selectivity models that assume all individual fish that enter the codend have been able to contact the codend meshes in such a way that corresponds to a size-dependent probability of escape [24]. These models are fully described by the size selectivity parameters length at 50% retention (L50) and selection range (length at 75% retention–length at 25% retention; SR), with one additional parameter (1/δ) for the Richard model. These models are described in Wileman et al. [41].

Another four models (Eq 3) are also considered that account for the percentage of fish that will not be able to make contact with the meshes in such a way that will lead to a size-dependent chance to escape [24]. These additional models have an additional parameter *C*, which represents the assumed length-independent contact probability of fish having contact with the codend meshes that corresponds to a length-dependent chance of escape. If *C* equals 1.0, then all fish had contact with the meshes to have a length-based size selectivity. If 0.75, then 75% of fish had contact with the meshes to lead to a length-based size selectivity. The last four considered models in Eq 3 have an additional subscript *c* which represents the inclusion of the percentage of fish that are actually estimated to make contact with the codend meshes that leads to length-based size selectivity. These models also have the overall L50 and SR consider which fish had sufficient contact, and are estimated based on the value of *C* [47]. Models that included contact probability parameters were considered to investigate whether a shaking codend actually improved the likelihood of redfish having contact with codend meshes in such a way that provides length-based size selectivity. It is plausible that a shaking codend could have more interaction with fish in the codend, either by physical contact only and/or by inducing escape behaviors that lead to contact.


$$r_{av}(l,v)=\left\{ \begin{array}{c} Logit(l,L50,SR)\\ Probit(l,L50,SR)\\ Gompertz(l,L50,SR)\\ Richard(l,L50,SR,1/\delta )\\ CLogit(l,C,{L50}_{c},{SR}_{c})=1.0-C+C\times Logit(l,{L50}_{c},{SR}_{c})\\ CProbit(l,C,{L50}_{c},{SR}_{c})=1.0-C+C\times Probit(l,{L50}_{c},{SR}_{c})\\ CGompertz(l,C,{L50}_{c},{SR}_{c})=1.0-C+C\times Gompertz(l,{L50}_{c},{SR}_{c})\\ CRichard(l,C,{L50}_{c},{SR}_{c},{1/\delta }_{c})=1.0-C+C\times Richard(l,{L50}_{c},{SR}_{c},{1/\delta }_{c}) \end{array}\right.$$ (3)

How the model fit the data was determined with a goodness-of-fit test described in Wileman et al. [41]. If the *p*-value was > 0.05, then the model was considered a good fit. If the *p*-value was < 0.05, then model residuals were invesitagated for structural problems. The best fit model was determined by the lowest AIC value.

Confidence intervals were produced using the double bootstrap method described in Millar [48] and Herrmann et al. [44]. The Efron percentile 95% confidence intervals (CIs; [49]) were fit for the best fit model with 1000 bootstraps.

Following Larsen et al. [50], the differences in size selectivity between the shaking and T90 codend were directly compared with a Delta plot using the *Δr(l)* function:

$$\Delta r(l)=r_{e}(l)–r_{c}(l)$$ (4)

where *r*_*e*_*(l)* and *r*_*c*_*(l)* are the size selectivity models for the shaking codend and the T90 codend, respectively. Confidence intervals were generated for *Δr(l)* from two bootstrap population results (Efron 95% confidence intervals from 1000 bootstraps each) for *r*_*e*_*(l)* and *r*_*c*_*(l)*. Since they were obtained independently, a new bootstrap population for *Δr(l)* was created using:

$$\Delta r{\left(l\right)}_{i}=r_{e}{\left(l\right)}_{i}-r_{c}{\left(l\right)}_{i}\;i\in [1\ldots 1000]$$ (5)

where *i* is the bootstrap repetition index. As explained in Moore et al. [51], since resampling was random and independent for both groups, it is valid to generate the bootstrap population of results for the difference based on two independently generated bootstrap groups using Eq 5. This approach will increase the power of inference between the shaking and T90 codend since the confidence limits for *Δr(l)* cannot go beyond those of *r*_*e*_*(l)* and *r*_*c*_*(l)*, and in general will often be smaller [50, 51]). Significant differences between codend size selectivity were determined by the location of CIs. If CIs do not overlap 0.0 at a particular length class then a significant difference is observed. However, if 0.0 is contained within the CIs then there is no difference in size selectivity between codends at the observed length class [52].

## Results

### Flume tank experiment

**Fig 3:**
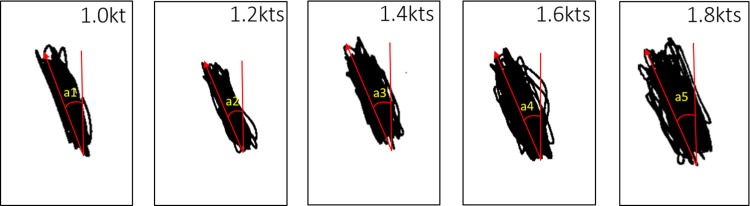
Movement of the shaking codend across five flow velocities from 1.0 to 1.8 kt. Black lines track codend movement angles (a1-a5) across 5 flow velocities, 1.0 kt, 1.2 kt, 1.4 kt, 1.6 kt, and 1.8 kt.

The moving angle of the shaking codend at each flow velocity measured in the flume tank is shown in Fig 3. The mean of the moving angle gradually increased from 21.0 (±0.08 SEM) to 22.6° (±0.15 SEM) as flow velocities increased from 1.0 to 1.8 kt (Table 1). The linear regression model for the relationship between the moving angle and flow velocity is Moving angle = 2.0085*Flow velocity + 19.131 (Table 1). Both the intercept and flow velocity parameters had positive slopes indicating that the mean moving angle of the shaking codend increases with increasing flow velocity (*p*-value < 0.001; Table 1).

**Table 1:** The mean moving angle of a shaking codend at each flow velocity and linear regression summary tested in a flume tank.

**Mean moving angle**				
**Flow velocity (kt)**	**Moving angle (°)**		**SEM**	
1.0	21.0		0.08	
1.2	21.6		0.16	
1.4	22.0		0.07	
1.6	22.5		0.12	
1.8	22.6		0.15	
**Linear regression summary**				
**Parameter**	**Estimate**	**SE**	***t* value**	***p* value**
(Intercept)	19.131	0.2824	67.75	**< 0.001**
Flow velocity	2.0085	0.1977	10.16	**< 0.001**

SEM is standard error of the mean and SE is the standard error. *p-*values in bold are statistically significant based on an alpha of 0.05.

**Fig 4:**
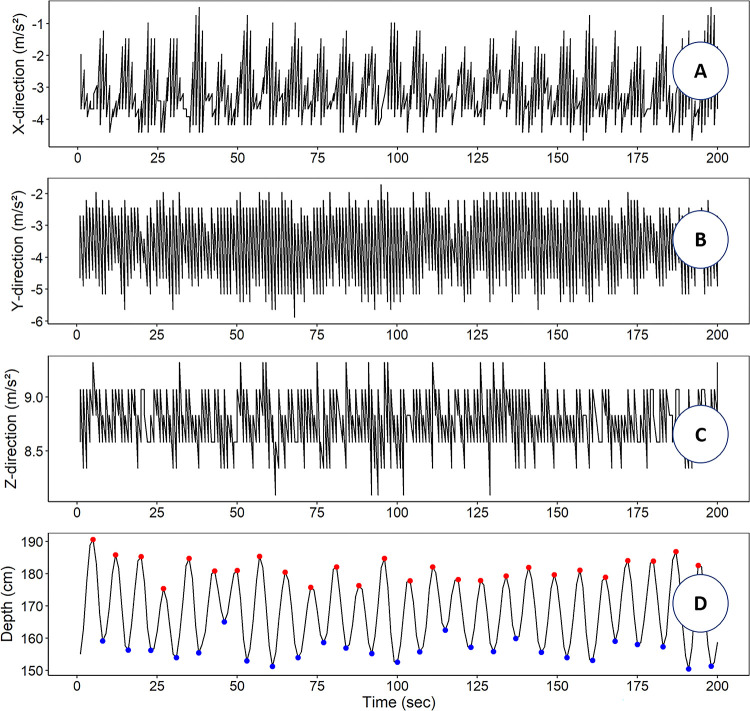
Examples of shaking codend acceleration and depth change in flume tank tests. The first three panels A, B and C are the acceleration values recorded in X, Y, Z directions, respectively. The D panel shows the results of the find_peak analysis applied for depth data to calculate the peak-to-peak amplitude; red points are the peaks and blue points are the valleys for the shaking codend. These examples were taken at a flow velocity of 1.8 kt over 200 secs.

Total acceleration, amplitude ratio, and period analysis began after 5 min from the beginning of each flow velocity test to allow the codend movement to stabilize. Thus, data for 25 min was recorded for each flow velocity iteration, except flow velocity 1.0 kt for the T90 codend, which was unexpectedly cut short at 9 min and 25 s. An example of one of these data sets is represented in Fig 4.

**Fig 5:**
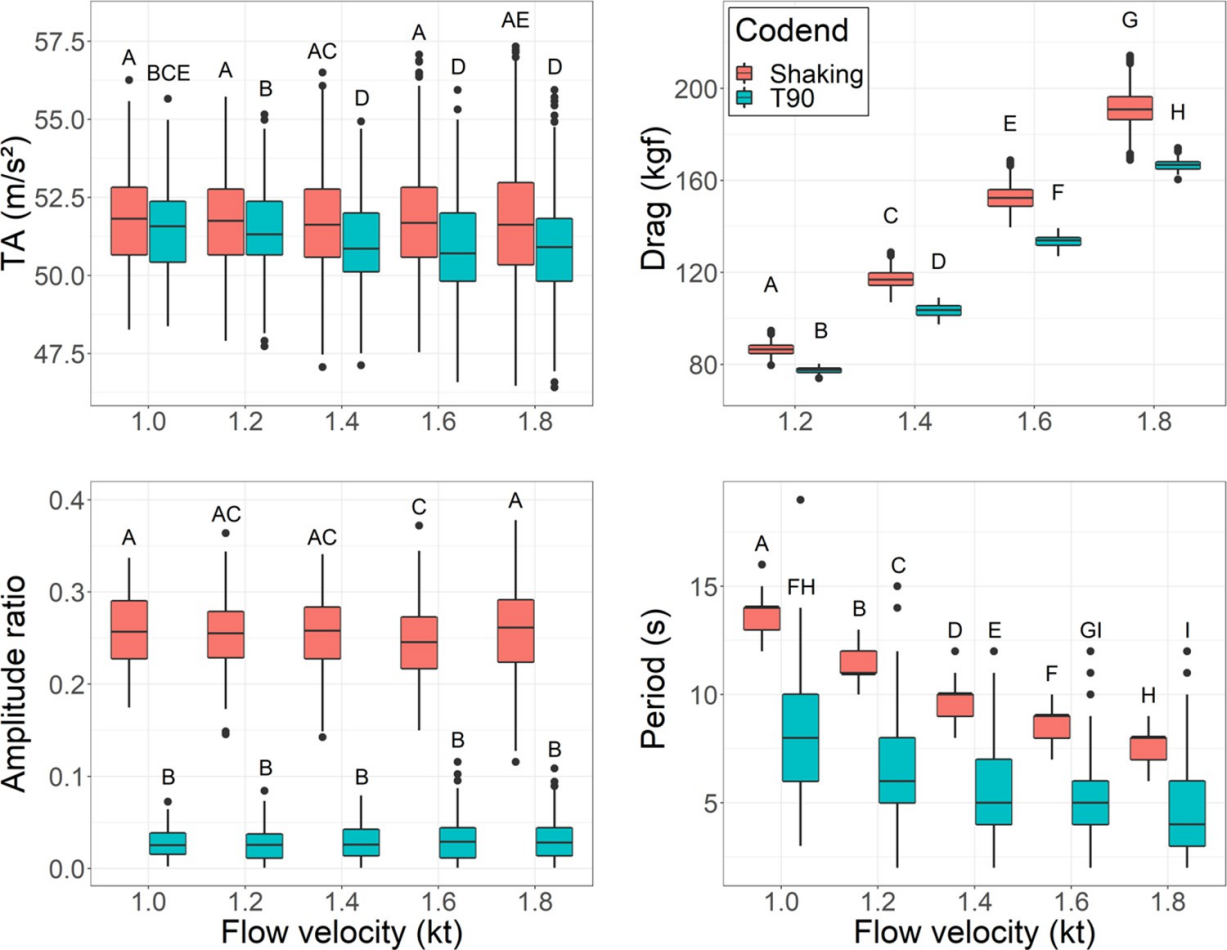
Boxplots of total acceleration, amplitude ratio, and period for codends evaluated in a flume tank test. The horizontal line in the middle of the boxes represents the 50^th^ percentile (median) the top and bottom limit of the boxes represents the 75^th^ percentile and 25^th^ percentile, respectively. Upper and lower whiskers are the 75^th^ (or 25^th^) percentile − 1.5 * interquartile range. Circles are values outside the range of the whiskers. Letters represent a significant difference between treatments (shaking or T90 codend) at a specific water velocity via *post hoc* analysis at an α of 0.05.

Mean TA trended higher for the shaking codend for each tested flow velocity and gradually reduced as flow velocity increased (Table 2) with the linear regression showing that the shaking codend had a higher overall TA (S1 Table). The best model contained codend, flow velocity, and their interaction term (S2 Table). A subsequent Tukey’s HSD post hoc test showed that the mean TAs generated by the shaking codend were significantly higher than those generated by the T90 codend at each corresponding flow velocity (*p*-value < 0.05; Fig 5). The mean TA of the shaking codend was not significantly different among flow velocities (*p*-value > 0.05; Fig 5). By comparison, the mean TA of the T90 codend was statistically higher at 1.0 and 1.2 kt compared to the other flow velocities (*p*-value < 0.001; Fig 5).

**Table 2:** The mean values for total acceleration (TA), amplitude ratio, and period of a shaking and T90 codend tested in five different flow velocities during a flume tank test.

Measurement	Codend	Flow velocity (kt)				
		1.0	1.2	1.4	1.6	1.8
TA (m s^-2^)	Shaking	51.84 (0.04)	51.75 (0.04)	51.68 (0.04)	51.78 (0.04)	51.69 (0.05)
	T90	51.50 (0.06)	51.38 (0.03)	51.06 (0.03)	50.90 (0.04)	50.92 (0.04)
Drag force (kgf)	Shaking	NA	86.60 (0.02)	117.12 (0.04)	152.73 (0.05)	191.50 (0.07)
	T90	NA	77.32 (0.04)	103.47 (0.07)	133.25 (0.1)	166.73 (0.08)
Amplitude ratio	Shaking	0.26 (0.004)	0.25 (0.003)	0.25 (0.003)	0.24 (0.42)	0.26 (0.004)
	T90	0.03 (0.002)	0.03 (0.001)	0.03 (0.001)	0.03 (0.001)	0.03 (0.001)
Period (s)	Shaking	13.63 (13.15–14.12)	11.36 (10.43–12.38)	9.80 (8.99–10.67)	8.52 (7.81–9.27)	7.59 (6.95–8.26)
	T90	8.29 (7.47–9.19)	6.92 (5.93–8.06)	5.96 (5.11–6.95)	5.18 (4.44–6.04)	4.62 (3.95–5.38)

The numbers in parentheses for TA and amplitude ratio are the standard error of the mean (SEM) values, and the numbers in parentheses for period are 95% confidence intervals.

The drag forces measured for the T90 codend at the flow velocity of 1.0 kt suffered a mechanical failure and were erroneous and were excluded from analysis. Thus, the difference in drag forces between the shaking and T90 codends was only compared at the flow velocities of 1.2–1.8 kt. The best model contained codend, flow velocity, and their interaction term (S1 Table). The model showed that the shaking codend had a higher overall drag force (S2 Table). A subsequent Tukey’s HSD post hoc test showed that the mean drag forces generated by the shaking codend were significantly higher than those generated by the T90 codend at each corresponding flow velocity (*p*-value < 0.001; Fig 5). The mean drag force of the shaking codend was significantly different among flow velocities and increased along with the flow velocity (*p*-value < 0.001; Fig 5). Though codend drag was higher for the shaking codend, it should be considered that codend drag is only a small proportion of total gear drag [53], and that the observed higher drag would only have a minimal increase in total drag and fuel consumption.

Recorded depths were used to generate amplitude ratio and period results, means are reported in Table 2. The best model for amplitude ratio included the codend, flow velocity, and their interaction term (S1 Table). The model showed that the amplitude ratios generated by the shaking codend were higher than those by the T90 codend (S2 Table). *Post hoc* analysis showed that for each tested flow velocity, amplitude ratio was higher for the shaking codend, slight but significant differences were observed between amplitude ratios for the shaking codend, and flow velocity did not affect the amplitude ratio for the T90 codend (Fig 5).

For period, the data were first fitted with a generalized linear model (GLM) using a Poisson link function. The dispersion of the model was determined to be underdispersed; thus, a quasi-Poisson link was used. The best model contained codend, flow velocity, and their interaction term (S1 Table), and showed that the T90 codend had a lower period (S2 Table). *Post hoc* analysis showed that the period of the shaking codend at each corresponding flow velocity was significantly higher than the T90 codend (*p*-value < 0.05; Fig 5). The period for each codend was highest at a flow velocity of 1.0 kt and gradually decreased, significantly, as flow velocity increased (*p*-value < 0.05; Fig 5), except the period for the T90 codend between 1.6 and 1.8 kt (*p*-value > 0.05; Fig 5).

### Sea trials experiment

A total of 15 hauls were completed, including 4 hauls for the shaking codend and 11 hauls for the T90 codend (T90 codend results originally reported in Cheng et al. [11]; Table 3). The mean depth of the fishing ground was 299.3 m (range: 234.1 to 329.2 m), the average haul duration was 7.8 min (range: 4 to 18 min), and the towing speed was between 2.3 and 2.6 kt (mean = 2.5 ± 0.08 standard deviation (SD)). The mean inside stretched codend mesh size for the T90 codend was 95.0 mm (SD = 2.4 mm) and 49.3 mm (SD = 1.9 mm) for the cover. The length of the warp ranged from 594.4 to 777.2 m (mean = 704.1 ± 38.7 SD), and the door spread was 66.2 m (range 63.4 to 68.9 m).

**Table 3:** Operational conditions for sea trials testing the size selectivity of shaking and T90 codends.

Codend	Haul ID	Date	Number of measurements		Subsampling ratio		Towing duration (min)	Maximum towing depth (m)	Haul velocity (kt)
			Cover	Codend	Cover	Codend			
Shaking	1	July 16, 2019	337	390	0.2643	0.0845	18	129	2.4
	2	July 16, 2019	371	199	0.8690	0.2701	10	129	2.5
	3	July 16, 2019	363	163	0.1974	0.0838	6	132	2.5
	4	July 16, 2019	369	285	0.6452	0.3432	6	140	2.4
T90	5	July 17, 2019	81	287	1	0.6435	5	179	2.4
	6	July 17, 2019	156	133	1	0.2036	9	180	2.3
	7	July 17, 2019	319	349	0.3734	0.0957	10	178	2.6
	8	July 17, 2019	358	358	0.1533	0.0567	9	177	2.5
	9	July 17, 2019	337	234	1	0.0871	7	178	2.5
	10	July 17, 2019	65	138	1	0.2108	7	176	2.5
	11	July 18, 2019	384	384	0.6540	0.1115	7	172	2.4
	12	July 18, 2019	407	362	0.4815	0.0975	8	172	2.4
	13	July 18, 2019	339	334	0.2018	0.0839	6	178	2.5
	14	July 18, 2019	328	364	0.3385	0.0707	5	177	2.6
	15	July 18, 2019	305	335	0.1860	0.0560	4	172	2.4

A total of 10250.1 kg redfish were captured during sea trials, 2784.7 kg by the shaking codend and 7465.4 kg by T90 codend. Of those, 8834 redfish were measured, 2477 for the shaking codend and covered codend versus 6357 for the T90 codend and covered codend (Table 3). The average redfish length was 22.7 cm (SEM = 1.5 cm) and ranged from 13–40 cm. Fishing depth changed over 0.5 m for several hauls, thus depth subsamples were applied to take the time intervals where the depth changed less than 0.5 m. There were 9 subsamples in total for three hauls covering the entirety of the time fishing (Table 4). The amplitude ratio ranged from 0.04 to 0.24, with a mean value of 0.11 (SD = 0.07). The mean for period was 9.2 s (SD = 4.92; range 4.0–20.7 s).

**Table 4:** Subsample number, amplitude ratio, and period of the shaking codend when tested during sea trials.

Haul ID	Subsampling	Amplitude ratio	Period (s)
2	1	0.13	7.7
	2	0.08	10.5
	3	0.14	9.1
	4	0.24	20.7
3	1	0.07	6.8
	2	0.05	4.6
	3	0.19	10.8
4	1	0.04	4
	2	0.08	8.2

**Fig 6:**
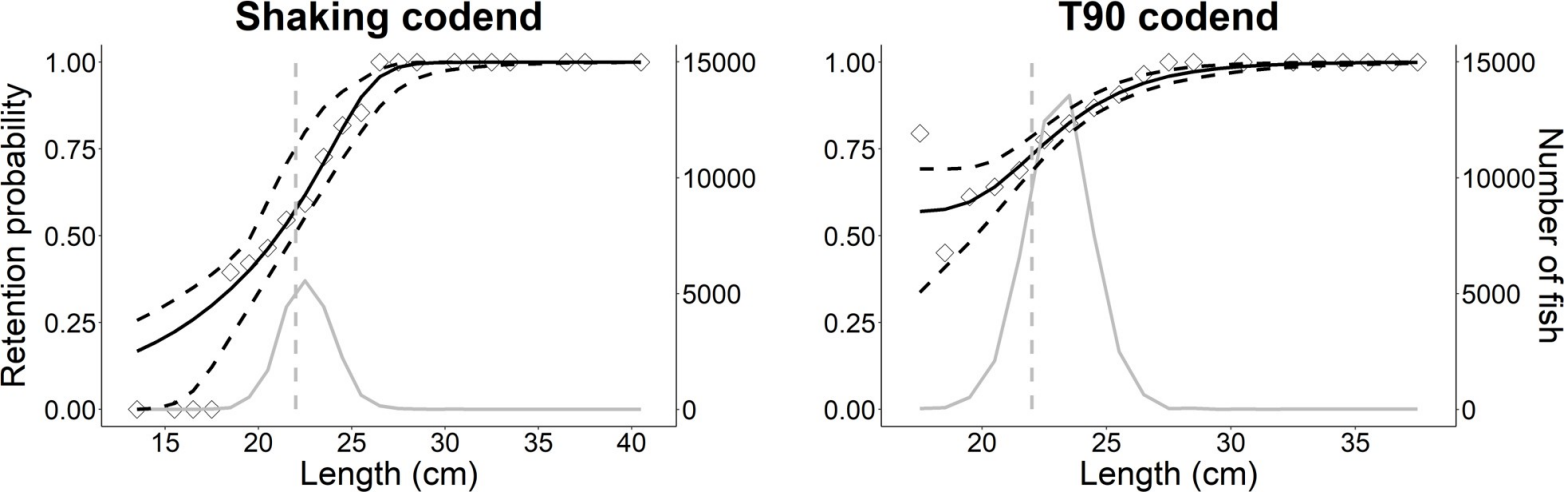
Size selectivity plots for the shaking and T90 codends. The black line represents the size selectivity curves. The vertical grey dashed lines represent the minimum landing size (MLS) for the Canadian redfish fishery. Diamonds correspond to the experimental ratios, whereas grey lines represent the size distribution of the redfish population captured during testing. Dashed black lines are the 95% Efron percentile confidence intervals.

**Fig 7:**
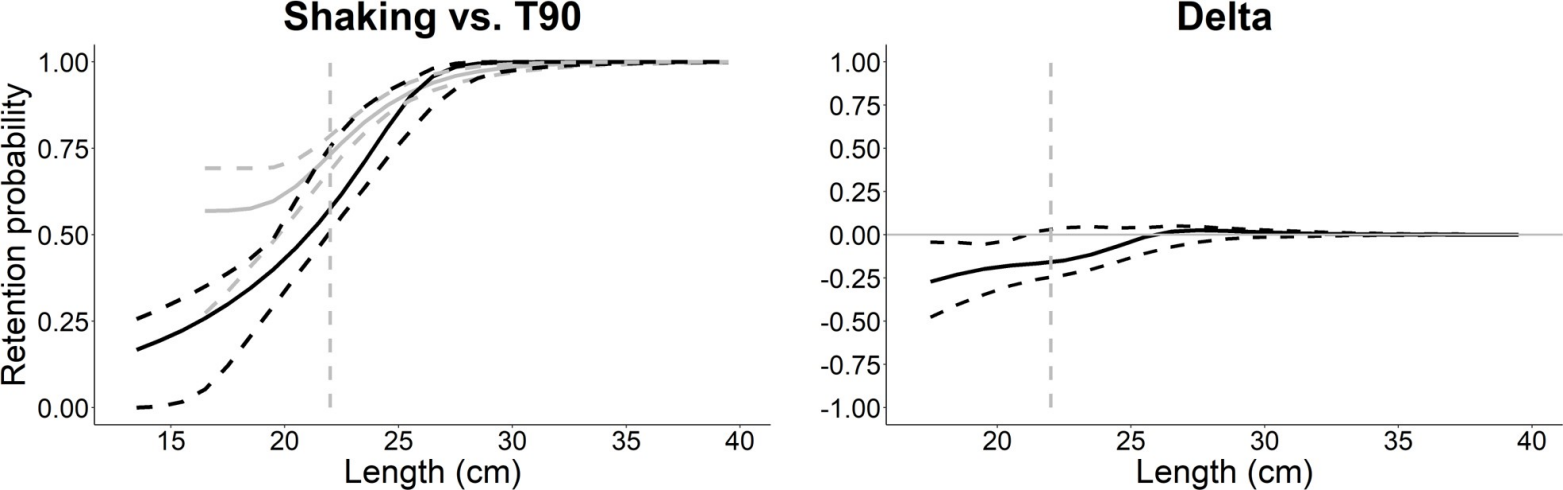
Selectivity comparison between the shaking and T90 codend. The left plot compares the size selectivity curves of the shaking codend versus the T90 codend: black and grey lines are the selection curves for the shaking codend and T90 codend, respectively; black and grey dashed lines are the 95% Efron percentile confidence intervals for the shaking and T90 codend, respectively. The right plot illustrates the delta curve: black line is the delta curve; grey dashed lines are the 95% Efron percentile confidence intervals. The vertical grey dashed lines in both plots represent the minimum landing size (MLS) for Canadian redfish fishery.

Based on the AIC values in Table 5, the Richard model was the best fit for the shaking codend. The size selectivity curve of the shaking codend showed lower retention for redfish < 21 cm (Figs 6 and 7). The L50 was 20.1 (CIs 19.6–21.9; Table 6) and SR could not be reasonably determined since only a few data points at the length at 25% retention (L25; SR is L75—L25) were observed (Fig 6); for similar examples see Cheng et al. [11], Einarsson et al. [45]. The model had a *p*-value < 0.05 likely due to overdispersion from the low number of hauls sampled. For the T90 codend, the CGompertz model was the best fit (Table 5). The L50 and SR could not be reported because they were not reached (Fig 6; [11, 45]). However, the L50_*c*_ was reached (22.6, CIs 20.0–23.8), which includes redfish that were able to make sufficient contact with the meshes that leads to size-dependent selection. The contact parameter, *C*, was 0.5 which indicates that 50% of redfish captured had appropriate contact with the meshes, however the confidence was over a large range of values (0.3–0.9; Table 6). The model did not suffer from overdispersion and had a *p*-value > 0.05 (Table 6).

**Table 5:** AIC values for fit size selectivity models.

Codend	Model							
	Logit	CLogit	Probit	CProbit	Gompertz	CGompertz	Richard	CRichard
Shaking	24,717.52	24,670.35	24,705.70	24,669.43	24,752.95	24,684.75	**24,663.26**	24,665.26
T90	45,281.88	45,257.56	45,269.85	45,256.59	45,302.46	**45,254.85**	45,260.14	45,257.23

Bold numbers specify the best models for each codend with the lowest AIC.

**Table 6:** Results from the best fit size selectivity models.

Codend	Shaking	T90
Model	Richard	CGompertz
L50	20.1 (19.6–21.9)	*
C	*	0.5 (0.3–0.9)
L50_*c*_	*	22.6 (20.0–23.8)
*p*-value	0.038	0.594
Deviance	31.26	14.06
DOF	19	16

Values in parentheses represent 95% confidence intervals. * is not applicable value.

## Discussion

This is the first known study to use a shaking codend at a commercial scale and to produce and compare size selectivity curves between a shaking and non-shaking codend. Our preliminary results obtanied during a small-scale sea trial indicated that the shaking codend reduced the capture of undersized redfish in Canada’s Gulf of St. Lawrence trawl fishery and was easy to handle and use in a commercial context. Flume tank tests showed that the shaking codend was much more dynamic than the T90 codend, having a larger amplitude ratio, period, and slightly larger TA and also had a higher drag. This movement likely led to the reduction in catch of small redfish, improving contact probability and potentially motivating fish to escape through the codend meshes.

The amplitude ratio for the shaking codend was significantly higher than the T90 codend during the flume tank test, and change in flow velocity showed minimal effects. However, sea trials showed a wide range of amplitude ratios, matching the highest observed in the tank test for the shaking codend (~0.25) to near the lowest observed for the T90 codend (~0.04). Many factors could have led to these results, perhaps with the main difference being the continuing changing depths found during the sea trials that can easily mask any change in amplitude recorded from a depth sensor. Other factors that could have led to these observed differences include changes in current, tow speed (max of 1.8 kt in flume tank and 2.6 kt during commercial fishing), and total catch. Simulated catch was constant in the tank tests but changed haul-to-haul during sea trials.

Similarly, period was significantly higher for the shaking codend when compared to the T90 codend during tank tests. However, during sea trials, wide ranges were also observed that were greater than the shaking codend (20.7 s) and lower than the T90 codend (4.0 s) during tank tests. Likely, this range is due to the factors discussed for the differences between amplitude ratios observed in the tank test and at sea, however period was observed to decrease in the tank test with increasing flow velocity. This would lead to an expectation that period at sea would be on the lower end of what was observed in the tank test (~4.0–7.5 s) since towing speeds were much higher. However, since period was measured to be over 20 s, it can be assumed that the codend movement was much more dynamic at sea.

Kim [19, 20] stated that a “shaking” ratio of 0.5 encouraged fish to move and approach codend nettings. Amplitude ratios in the reported study stayed below 0.5, though preliminary results of size selectivity suggest that escapes could have been increased; no video observations were made to determine if these escapes were derived from behavioural, mechanical, or both selectivity mechanisms. However, comparing the amplitude ratio between our study and Kim [19, 20]’s should not necessarily be considered one-to-one in terms of the specific amplitude ratio value. Kim [19, 20] used metal rings at the end of his codend that the tarp was placed over. The amplitude ratio was based on the diameter of the tarp. This is reasonably similar to what would be expected of the shape of a 2-panel codend, round and bulbous shape, but our study used a 4-panel codend that is not bulbous at the end, as each panel comes to a point at the terminal end. Additionally, differences in how this study and Kim [19, 20]’s accounted for distances relative to the gear construction could have led to subtle differences in amplitude ratios between the studies.

Beyond differences in study design between the reported study and Kim [19, 20], Kim [19, 20] used traditional codend netting (T0) where we used T90. Hansen [54] and Madsen et al. [55] showed that the T0 codend’s movements are much more dynamic than the T90 codend. The movement of a codend is generally forced by the turbulence intensity inside the codend [54]. Water flow through the codend is lower in a T0 codend, versus a T90 codend, due to the mesh openings remaining more closed under the load of the catch and restricting the water flow [30]. This reduction in flow likely increases the turbulence and leads to increased movement. Additionally, since Kim [19, 20] used a trawl more closely resembling a 2-panel codend, and we used a 4-panel, it is reasonable to assume that the addition of two more riblines would also produce a more stable codend. Thus, the characteristics of a T90, 4-panel codend has inherit characteristics that may reduce its movement capacity when compared to 2-panel and T0 codends.

Kim [19] tested a shaking codend at sea, but this was to document the movements only and consisted of the codend attached to a towing frame and opened by metal hoops, not an actual trawl attached to a vessel with warps, spread with doors, etc. Here, we were able to test a shaking codend on a commercial fishing vessel and not only quantify its movement and size selectivity, but assess how such a design could be used in a commercial setting. Certainly, adding a tarp over the codend adds time in terms of opening and closing the codend, since the tarp is overtop of the typical location of the codend opening. However, we were able to use twine to fasten the tarp to the codend, which took approximately 20 min. If such a design was used during commercial fishing, improvements should be considered to shorten the time to take the tarp on-and-off to have access to the codend opening. Using a more fast-opening process, such as a large, heavy-duty zipper [56], instead of tying the tarp on-and-off for each haul could be a more time-efficient approach to be used in commercial fisheries. Otherwise, the addition of the tarp was found to have no other observed effect on the handling of the fishing gear.

Smaller redfish were shown to be retained less often by the shaking codend and this may be explained by the improved contact probability of the shaking codend. For example, we could not report the L50 for the T90 codend because fish that small were not captured (these size classes were not present in the fishery [7]), but when only considering fish that had appropriate contact with the codend meshes (L50_*c*_), the L50 and L50_*c*_ were not significantly different between the shaking and T90 codend. This suggests that what is driving the significant difference between the shaking and T90 codend are small redfish that are not having appropriate contact with codend meshes that leads to size dependent selectivity. Likely, these fish are not able to reach the codend meshes due to congregation of redfish within the codend.

What is causing redfish to have improved contact in the shaking codend is not clear. The shaking codend could simply be providing more physical contact to fish that are not very active in the codend, i.e. the movement leads to more fish having contact with the meshes. Conversely, the shaking codend could be promoting behaviour (e.g. escape attempts) that leads to more contact with the codend. Active stimulation devices have been shown to increase small fish escape [16, 17, 19, 20], which likely could improve the contact probability between fish and the codend meshes. The sweeping of upper and lower panels when the codend moves vertically could reduce the space in the codend [20] that can reduce the available swimming space of fish in the codend, as well as the distance between the meshes and the fish. These effects can lead to an increase in fish contact with the meshes and penetration through the meshes, rather than being impinged to the codend and prevented from escape [57]. Overall, the escape probability of fish through the codend meshes is related to the distance between fish in the middle of the codend and codend panels. This distance likely can be decreased with increasing amplitude ratio (overall minimum distance to codend panels reduced on average), leading to more fish in the codend come into contact with the meshes, and therefore increasing escape likelihood. Future work should consider using cameras to document redfish behaviour to a shaking codend.

Fish escape through codend meshes can be affected by swimming endurance, which is generally limited once fish have reached the codend [14]. Additionally, turbulence generated from the rear of the codend can also affect swimming endurance by reducing swimming speed required to maintain station ahead of accumulated catch [14, 58]. Likely, the relatively high turbulence created by attaching the canvas at the rear of the codend in this study, combined with the movement of the codend, potentially provide redfish an additional capacity to keep station ahead of accumulated catch and orient to swim through codend meshes [14]. This could lead to a relatively higher number of redfish, particular small individuals, to come into contact with the codend meshes to escape, compared with the codend without canvas.

There is some disparity between the number of hauls for the shaking codend and the T90 codend. The data collected for the T90 codend was from a previously published [11], separate study comparing the size selectivity between three T90 codends (90, 100, 110 mm mesh size) and the traditionally used codend (90 mm T0 mesh). Though this was a separate study, the hauls for each codend reported in this study were performed on consecutive days. Thus, we took the opportunity to compare the size selectivity between a shaking codend and a well performing experimental codend (T90, 90 mm mesh size) that isolates the variable of interest (i.e. shaking codend) to quantify its size selectivity performance. Further, the number of replicates reported for the shaking codend, *n* = 4, is relatively small, but not out of line from recent size selectivity publications, Ingólfsson and Brinkhof [59]; *n* = 5 per treatment and Petetta et al. [60]; *n* = 6 per treatment. Additionally, the Ingólfsson and Brinkhof [59] study has a relative selectivity study design, which has a much lower statistical power [61], making the statistical inference at a low haul number much more challenging than for a covered codend design (as used in the reported study), where escaped fish sizes are measured. Nevertheless, the difference in replicates between treatments should provide some caution, and further research should continue to fully understand the size selectivity of a shaking codend. That said, both the model fit and confidence interval size shows that the data collected are reasonable for the applied analyses.

In conclusion, our experiment developed a full-scale shaking codend from a 90 mm T90 codend, which has been suggested to replace the currently regulated 90 mm T0 to improve the size selectivity of the Gulf of St. Lawrence redfish fishery [11]. Currently, the Gulf of St. Lawrence has a large biomass of deepwater redfish and a commercial fishery is imminent [7]. Our preliminary results from the sea trials show that a shaking codend further reduced the capture of small fish than the T90 codend alone, which already showed great improvement when compared to the traditionally used T0 codend [11]. The development of new methods to sustainably harvest redfish are necessary to prevent overfishing of a species that is sensitive to fishing. In this study, the shaking codend has shown potential to be used to harvest redfish sustainably, capturing fewer undersized fish.

## Supporting information

S1 Table Flume tank experiment analyses comparing the total acceleration, drag forces, amplitude ratio, and period between a shaking codend and T90 codend. (DOCX)

S2 Table Summary of total acceleration linear regression, drag force linear regression, amplitude ratio linear regression, and period generalized linear model comparing total acceleration, amplitude ratio, and period between a shaking and T90 codend during a flume tank test. (DOCX)

S1 Data (ZIP)
