# Supplementary material for: Developing a full-scale shaking codend to reduce the capture of small fish
Source: PLoS One. 2023 Jan 23;18(1):e0280751. doi: 10.1371/journal.pone.0280751 (PMC9870171; doi:10.1371/journal.pone.0280751)
Supplement: S1 Table — (DOCX) [file pone.0280751.s001.docx]

**S1 Table.** Flume tank experiment analyses comparing the total acceleration, drag forces, amplitude ratio, and period between a shaking codend and T90 codend.

| **Total acceleration** |  |  |  |
| --- | --- | --- | --- |
| Independent variables | AICc | dAICc | weight |
| Codend * Flow velocity | **51253.0** | 0 | 1 |
| Codend + Flow velocity | 51299.4 | 46.5 | <0.001 |
| Codend | 51368.7 | 115.7 | <0.001 |
| Flow velocity | 51873.0 | 620.0 | <0.001 |
| Null model | 51992.6 | 739.6 | <0.001 |
| **Drag force** | | | |
| Independent variables | AICc | dAICc | weight |
| Codend * Flow velocity | 283676.7 | 0.0 | 1 |
| Codend + Flow velocity | 287503.5 | 3826.8 | <0.001 |
| Codend | 309048.7 | 25372.0 | <0.001 |
| Flow velocity | 466534.3 | 182857.6 | <0.001 |
| Null model | 467448.7 | 183772.0 | <0.001 |
| **Amplitude ratio** |  |  |  |
| Independent variables | AICc | dAICc | weight |
| Codend * Flow velocity | **-8044.8** | 0 | 0.979 |
| Codend | -8035.7 | 9.1 | 0.010 |
| Codend + Flow velocity | -8035.6 | 9.2 | 0.009 |
| Flow velocity | -3002.9 | 5041.9 | <0.001 |
| Null model | -2967.0 | 5077.9 | <0.001 |
| **Period** |  |  |  |
| Independent variables | QAIC | dQAIC | weight |
| Codend * Flow velocity | **16837.1** | 0 | 1 |
| Codend + Flow velocity | 16841.4 | 4.3 | 0.11 |
| Codend | 17871 | 1027.3 | <0.001 |
| Flow velocity | 18820.1 | 1976.4 | <0.001 |
| Null model | 20221.5 | 3377.8 | <0.001 |

Independent variables included in models, Akaike information criterion (AICc) or QAIC, delta-AICc (dAICc) or dQAIC, and AICc/QAIC weight (weight) for each model. Bold numbers specify the best model with the lowest AICc or QAIC.
