# Supplementary material for: Developing a full-scale shaking codend to reduce the capture of small fish
Source: PLoS One. 2023 Jan 23;18(1):e0280751. doi: 10.1371/journal.pone.0280751 (PMC9870171; doi:10.1371/journal.pone.0280751)
Supplement: S2 Table — (DOCX) [file pone.0280751.s002.docx]

**S2 Table.** Summary of total acceleration linear regression, drag force linear regression, amplitude ratio linear regression, and period generalized linear model comparing total acceleration, amplitude ratio, and period between a shaking and T90 codend during a flume tank test.

| **Total acceleration** | | | | |
| --- | --- | --- | --- | --- |
| Variable | Estimate | SE | *t* value | *p* value |
| (Intercept) | 51.836 | 0.038 | 1331.059 | **< 0.001** |
| CodendT90 | -0.331 | 0.078 | -4.251 | **< 0.001** |
| Flow velocity 1.2 kt | -0.085 | 0.055 | -1.545 | 0.122 |
| Flow velocity 1.4 kt | -0.153 | 0.055 | -2.787 | **0.005** |
| Flow velocity 1.6 kt | -0.052 | 0.055 | -0.953 | 0.34 |
| Flow velocity 1.8 | -0.142 | 0.055 | -2.594 | **0.009** |
| Codend T90:Flow velocity 1.2 kt | -0.037 | 0.095 | -0.39 | 0.696 |
| Codend T90:Flow velocity 1.4 kt | -0.292 | 0.095 | -3.06 | **0.002** |
| Codend T90:Flow velocity 1.6 kt | -0.550 | 0.095 | -5.764 | **< 0.001** |
| Codend T90:Flow velocity 1.8 kt | -0.439 | 0.095 | -4.597 | **< 0.001** |
| **Drag force** | | | | |
| Variable | Estimate | SE | *t* value | *p* value |
| (Intercept) | 86.60321 | 0.0477 | 1815.6 | **< 0.001** |
| Codend T90 | -9.28177 | 0.16225 | -57.21 | **< 0.001** |
| Flow velocity 1.4 kt | 30.5206 | 0.06739 | 452.92 | **< 0.001** |
| Flow velocity 1.6 kt | 66.12564 | 0.06802 | 972.21 | **< 0.001** |
| Flow velocity 1.8 kt | 104.9 | 0.06755 | 1552.86 | **< 0.001** |
| Codend T90:Flow velocity 1.4 kt | -4.36802 | 0.22366 | -19.53 | **< 0.001** |
| Codend T90:Flow velocity 1.6 kt | -10.196 | 0.26232 | -38.87 | **< 0.001** |
| Codend T90:Flow velocity 1.8 kt | -15.4948 | 0.23945 | -64.71 | **< 0.001** |
| **Amplitude ratio** |  |  |  |  |
| Variable | Estimate | SE | *t* value | *p* value |
| (Intercept) | 0.257 | 0.002 | 87.249 | **< 0.001** |
| Codend T90 | -0.229 | 0.004 | -48.081 | **< 0.001** |
| Flow velocity 1.2 kt | -0.003 | 0.004 | -0.857 | 0.391 |
| Flow velocity 1.4 kt | -0.002 | 0.003 | -0.694 | 0.487 |
| Flow velocity 1.6 kt | -0.013 | 0.003 | -3.542 | **< 0.001** |
| Flow velocity 1.8 kt | -0.001 | 0.003 | -0.114 | 0.908 |
| Codend T90:Flow velocity 1.2 kt | 0.001 | 0.005 | 0.32 | 0.748 |
| Codend T90:Flow velocity 1.4 kt | 0.004 | 0.005 | 0.778 | 0.436 |
| Codend T90:Flow velocity 1.6 kt | 0.016 | 0.005 | 2.987 | 0.002 |
| Codend T90:Flow velocity 1.8 kt | 0.003 | 0.005 | 0.687 | 0.492 |
| **Period** |  |  |  |  |
| Variable | Estimate | SE | *t* value | *p* value |
| (Intercept) | 2.612 | 0.018 | 144.228 | **< 0.001** |
| Codend T90 | -0.496 | 0.034 | -14.301 | **< 0.001** |
| Flow velocity 1.2 kt | -0.180 | 0.025 | -7.054 | **< 0.001** |
| Flow velocity 1.4 kt | -0.329 | 0.025 | -12.844 | **< 0.001** |
| Flow velocity 1.6 kt | -0.469 | 0.025 | -18.343 | **< 0.001** |
| Flow velocity 1.8 kt | -0.585 | 0.025 | -22.861 | **< 0.001** |
| Codend T90:Flow velocity 1.2 kt | -0.063 | 0.043 | -1.469 | 0.142 |
| Codend T90:Flow velocity 1.4 kt | -0.052 | 0.043 | -1.225 | 0.221 |
| Codend T90:Flow velocity 1.6 kt | -0.052 | 0.043 | -1.218 | 0.224 |
| Codend T90:Flow velocity 1.8 kt | -0.007 | 0.043 | -0.169 | 0.866 |

SE is the standard error and the *p* values in bold are statistically significant based on an alpha of 0.05.
